# How can we improve the experiences of patients and families who request medical assistance in dying? A multi-centre qualitative study

**DOI:** 10.1186/s12904-021-00882-4

**Published:** 2021-12-08

**Authors:** Simon J. W. Oczkowski, Diane E. Crawshaw, Peggy Austin, Donald Versluis, Gaelen Kalles-Chan, Michael Kekewich, Dorothyann Curran, Paul Miller, Michaela Kelly, Ellen Wiebe, Andrea Frolic

**Affiliations:** 1 Department of Medicine, McMaster University, Juravinski Hospital Room A3-20, 711 Concession St., Hamilton, ON L8V 1C1 Canada; 2 Department of Health Research Methods, Evidence, and Impact, McMaster University, Hamilton, Canada; 3 Hamilton Health Sciences, Hamilton, Canada; 4 Vancouver Island Health Authority, Victoria, Canada; 5 Department of Clinical and Organizational Ethics, The Ottawa Hospital, Ottawa, Canada; 6 The Ottawa Hospital, Ottawa, Canada; 7 Division of Emergency Medicine, Department of Medicine, McMaster University, Hamilton, Canada; 8 London School of Hygiene and Tropical Medicine, London, UK; 9 Department of Family Medicine, University of British Columbia, Vancouver, Canada; 10 Department of Family Medicine, McMaster University, Hamilton, Canada

**Keywords:** Euthanasia, Suicide, assisted, Quality of care, Patient-focused research, Qualitative research

## Abstract

**Background:**

Medical assistance in dying has been available in Canada for 5 years, but it is unclear which practices contribute to high-quality care. We aimed to describe patient and family perspectives of quality of care for medical assistance in dying.

**Methods:**

We conducted a multi-centre, qualitative descriptive study, including face to face or virtual one-hour interviews using a semi-structured guide. We interviewed 21 english-speaking patients found eligible for medical assistance in dying and 17 family members at four sites in Canada, between November 2017 and September 2019. Interviews were de-identified, and analyzed in an iterative process of thematic analysis.

**Results:**

We identified 18 themes. Sixteen themes were related to a single step in the process of medical assistance in dying (MAID requests, MAID assessments, preparation for dying, death and aftercare). Two themes (coordination and patient-centred care) were theme consistently across multiple steps in the MAID process. From these themes, alongside participant recommendations, we developed clinical practice suggestions which can guide care.

**Conclusions:**

Patients and families identified process-specific successes and challenges during the process of medical assistance in dying. Most importantly, they identified the need for care coordination and a patient-centred approach as central to high-quality care. More research is required to characterize which aspects of care most influence patient and family satisfaction.

**Supplementary Information:**

The online version contains supplementary material available at 10.1186/s12904-021-00882-4.

## Introduction

Since the passing of Bill C-14 in 2016, over 13,946 Canadians have received medical assistance in dying (MAID), representing approximately 2% of all deaths [1]. Bill C-14 legalized assisted dying for capable adult patients, with intolerable suffering whose death is “reasonably foreseeable,” and specified procedural safeguards, including the need for two witnesses of a written request for MAID; a 10 day waiting period; and confirmation of patient’s right to withdraw request, including immediately prior to MAID. While Bill C-14 detailed criteria by which a patient could become eligible for MAID, it provided little detail on how MAID should be implemented in clinical practice, and as a result it allows both assisted suicide (in which a patient self-administers a fatal dose of medication) and voluntary euthanasia (in which a clinician administers a fatal dose) [2, 3]. Though the MAID process has a straightforward technical outcome (a rapid and painless death), understanding whether or not the process leading to that outcome has been of high quality is subjective, and must be understood by exploring the experiences and perceptions of those who take part, particularly patients and families, as described in a recent systematic review [4]. While new Canadian Federal reporting requirements for MAID took effect in 2018, the collected data is broadly descriptive, and little to no in-depth data on the patient and family member experiences of MAID to guide clinical practice and guide research [1]. To address this gap, we conducted an exploratory qualitative study to understand patient, family, and clinician perspectives on quality of MAID care.

## Methods

### Setting and participants

The research team use a pre-specified protocol to guide study procedures, including recruitment, eligibility, interview process, and analysis. We conducted interviews with MAID-eligible patients, their families, and health care providers at four Canadian centres, two academic hospital-based with interdisciplinary teams (Hamilton and Ottawa), one a community hospital program lead by nurse practitioners (Niagara) and one an outpatient clinic (Vancouver). This manuscript includes patient and family descriptions of the quality of care during the MAID process. We reported HCP interviews separately as these described clinician-specific operational and institutional aspects of MAID care, such as remuneration, scheduling, and institutional supports which would only indirectly impact patient and family experiences [5].

After approval by the research ethics board (HIREB 3991 November 2017), clinicians at participated study sites asked MAID patients if they would be willing to be approached for research. If so, we invited patients who had been found eligible for MAID to participate in the study. If they agreed to take part, we subsequently invited one family member (as chosen by the patient who could be biologically related or not) and health care provider (preferentially one of the MAID assessors or providers, though we also included nurses, social workers, and managers who were directly involved in the patient’s MAID process) to participate as well. At least two of the three potential participants had to provide documented written or verbal consent to an interview to be included in the study. The only exclusion criteria was if a potential participant was unable to speak and read English. Our purposive sampling strategy was “typical” case sampling, aiming to recruit people who could provide experiential knowledge across the depth and breadth of the most common MAID experiences, as opposed to purposely recruiting ‘extraordinary’ or ‘unusual’ cases.

### Data collection

We developed a semi-structured interview guide which aimed to elicit participant perspectives of quality of care throughout the MAID process, and sufficiently general to apply across settings and a variety of care models. The guide was developed by the research team (including both non-clinician researchers and MAID clinicians), and asked participants about their experiences, facilitators and barriers to accessing MAID, and perceptions of what made for a positive MAID experience. The interview guide was updated iteratively during the study design and REB approvals process, however only minor wording changes were made after the first interview, as it appeared to elicit a broad range of experiences sufficient for our analysis (Supplementary Material). Investigators at each site without a relationship to the participant (DEC, SO, DC, MK) conducted and recorded interviews in person or by phone as per participant’s preference. Patients were interviewed shortly after study enrolment, while families were interviewed at a time of their choice after the patient’s death, usually 4–8 weeks later. At the time of interviews, participants also completed a basic demographic form. Recordings were transcribed, de-identified, and uploaded to Dedoose, a cloud-based qualitative data management program, for organization and analysis [6]. To allow for adequate breadth and depth of data, the target sample size was 60 interviews, including 20 patients, 20 families, and 20 HCPs, which we estimated would allow us to reach thematic saturation [7, 8].

### Data analysis

Aiming to provide high descriptive and interpretive validity, so as to identify practical improvements to the MAID clinical process, we used a qualitative descriptive approach of interview data. Given the legislative requirements for MAID and their chronology (MAID request; MAID assessments; preparation for dying; death and aftercare) we organized codes and themes according to this sequence [9, 10]. The primary analysis team (SO,DEC, PA) conducted open coding on the first three interviews to develop an initial codebook, including rules for when specific codes should be applied to the data. Analysis continued alongside participant recruitment and interviews, with the team meeting periodically to review new interview results and revise the codebook as new findings emerged. In an iterative process of thematic analysis, we developed primary and secondary themes related to perceptions of quality of care within our theoretical chronology of the MAID process [11]. To assist with knowledge translation of these findings, we developed practice suggestions from two sources, either direct study participant recommendations, or inferences based upon participant descriptions of successes and challenges in the care process [12]. As the study participants were either deceased or grieving, we did not use member-checking or allow for participant review of transcripts.

We incorporated several strategies to increase trustworthiness of our results [13]. Confirmability is enhanced by its use of a pre-specified protocol, interview guide, and adherence to reporting guidelines (COREQ); credibility by its use of multiple interviewers including clinicians and non-clinicians for triangulation during analysis, as well as its descriptive analytical approach; and transferability and dependability applicability by its multi-centre recruitment strategy; inclusion of hospital and the community settings.

## Results

Between November 2017 and September 2019, we recruited 24 patients and 17 family members to participate in the study. Twenty-one patients and all 17 family members completed an interview, meeting our goals for thematic saturation (identifying no new codes or themes) with fewer families than anticipated. In all 17 family interviews, the corresponding patient was interviewed; in the four remaining interviews, the patient and a HCP (but not the family) provided interviews. In one case, the patient died before interviews were completed; in the others, only the HCP completed an interview. Participant demographics are reported in Table 1. Similar to general demographics of Canadian MAID patients, cancer was the most common qualifying condition for MAID (*n* = 15, 63%), followed by cardiac or respiratory disease (*n* = 4 17%) and neurologic disease (*n* = 3, 13%). Two patients (8%) had other conditions. The patients all had significant functional limitation, with Eastern Cooperative Oncology Group grade of 2 (*n* = 3, 13%), 3 (*n* = 8, 33%), or 4 (*n* = 8, 33%) [14]. In contrast to patients, who were roughly evenly divided between genders (*n* = 11, 46% female; *n* = 13, 54% male), family members who reported gender were most often female (*n* = 13, 76%).

**Table 1:** Participant characteristics

**Patient characteristics (*n*** **= 24)**	
Gender, n(%)	Female 11 (46%) Male 13 (54%)
Age Range, n(%)	41–50, 1 (4%) 51–60, 4 (17%) 61–70, 6 (25%) 71–80, 5 (21%) 81–90, 5 (21%) > = 91, 3 (12%)
Diagnoses, n(%)	Cancer 15 (63%) Amyotrophic lateral sclerosis 3 (12%) Cardiovascular/Respiratory 4 (17%) Other neurological 1 (4%) Other (hotel) 1 (4%)
Current location, n(%)	Hospital 18 (75%) Home 5 (21%) Other 1 (4%)
European Cooperative Oncology Group Functional Status, n(%)	0, 1: 0 2: 3 (12%) 3: 8 (32%) 4: 8 (32%) Unreported/unsure: 5 (20%)
Preferred location of assisted dying, n(%)	Home 6 (24%) Hospice 1 (4%) Hospital 12 (50%) No preference 3 (12%) Other 2 (18%)
Preferred route of medications for assisted dying, n(%)	Oral medications 1 (4%) Intravenous 17 (71%) No preference 6 (25%)
Preferred timing of assisted dying, n(%)	Within one week 10 (42%) One week to one month 4 (17%) Greater than one month 5 (21%) No preference 5 (21%)
**Family member characteristics (*n*** **= 17)**	
Gender, n(%)	Male 3 (18%) Female 13 (76%) Declined to answer 1 (6%)
Age Range, n(%)	21–30, 1 (6%) 31–40, 0 41–50, 1 (6%) 51–60, 3 (18%) 61–70, 9 (53%) 71–80, 2 (12%) 81–90, 1 (6%)
Location, n(%)	Ontario, 15 (88%) British Columbia, 1 (6%) Nova Scotia, 1 (6%)
Relationship to patient, n(%)	Spouse/partner 6 (35%) Child 7 (41%) Sibling 2 (12%) Friend 2 (12%)

**Fig. 1:**
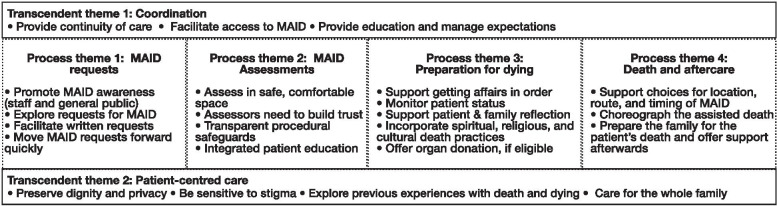
Study themes and subthemes

We organized themes related to perceptions of quality of care as either “process” themes related to a specific step of the MAID process as outlined in the interview guide (MAID request; MAID assessment; preparation for death; death and aftercare), or as a “transcendent” theme which which themed across multiple steps in MAID process (Fig. 1). Below, we summarize these major themes, and provide exemplar quotes. A complete list of themes, sub-themes, and exemplar quotes can be found in Supplementary Material 2. We summarize the practice suggestions in Table 2.

**Table 2:** Summary of themes and associated practice suggestions

Theme/Subtheme	Suggested practices
***Process Theme 1: MAID requests***	
Encourage MAID awareness	• Hospitals and clinics which care for patients at high risk of death should have publicly available information about MAID so patients are aware of this option • Awareness efforts should target the general public and families to help prepare them for the possibility their loved ones may choose MAID • Institutions should have clear policies around HCPs bringing up MAID
Explore requests for MAID	• HCPs should be aware that patients may struggle to bring up MAID, even if they are seriously considering it • Support HCPs in how to recognize and explore requests for a hastened death • Use direct language to acknowledge and explore a patient’s request for MAID
Facilitate written requests	• Written requests and other paperwork need to be simple and accessible • Assist with completing the written requests and witnessing, to avoid the need for corrections/revisions, and consequent delays in care • Provide contact information for Dying with Dignity which can provide volunteer witnesses, or have institutional volunteers who can assist inpatients
Move MAID requests forward quickly	• Have clear institutional policy/protocol for referring patients who request MAID • Policies should be supportive of conscientious objectors but mandate rapid referral/transfer of patients so as not impede timely response to MAID requests • Provide a map MAID process to patients so they can keep track of next steps and hold the team accountable for moving the request forward
***Process Theme 2: MAID Assessments***	
Assess in a safe, comfortable space	• Provide an outline of the purpose and content of the assessment • Identify private spaces for assessments, ask the patient where they would like to meet and who they would like to have present • Consider use of videoconferencing/telemedicine, as traveling for an assessment may be a challenge for some patients
Assessors need to build trust	• Introduce clinicians and their role in the MAID process • Acknowledge the need to build trust, if this is a new relationship • Frame the assessment as a conversation; explain the legal criteria for MAID • Use a conversational manner rather than “checklist” approach to encourage information sharing • Facilitate involvement of clinicians the patient has an existing relationships with (eg. family physician) in the assessment process
Provide transparent procedural safeguards	• Reinforce that the patient can change their mind or stop the process at any time • Having a portion of the assessment with and without family present to ensure non-coercion; be transparent about the reasons for this • Having two HCPs present for each assessment (eg MD/NP with an allied health member) can be a safeguard for both the patient and HCP
Provide education	• If the patient is willing, encourage close family members to be present during the assessment, so they can understand the patient’s motivations for MAID • Use the assessment as an opportunity to educate and prepare patients and families; MAID assessors and providers often have the best information on what to expect and prepare for
***Process theme 3: Preparation for dying***	
Getting affairs in order	• Develop a list of tasks to be completed, including estate planning • Involve social workers to assist in these decisions and processes as some families may find making arrangements cathartic while for others is may be burdensome
Support patient and family reflection	• Prepare patients and families for the emotional nature of the waiting period and acknowledging that complexity of emotions they may be experiencing • Ask and explore what families and patients need to make the most out of the waiting period • Specialist palliative care consultation can provide valuable support
Monitor patient status	• Identify patients at risk of losing capacity early, and ask patients/families if this is a source of anxiety for them • Explain available options and assess preferences in the event that capacity is lost • For patients at high risk of losing capacity, consider close monitoring or expediting assessments and consent process • Offer to reassess patients who have lost capacity as this may fluctuate • Discuss with patients palliative care options which may best preserve capacity, if MAID is their overriding preference
Incorporate patients’ spiritual and cultural death practices	• Explore preferences for spiritual/religious counselling is important regardless of stated religious followings • It may be helpful to identify faith leaders from various religions who are supportive of MAID and can “step in” to the role of the patient/family’s usual faith community • Non-religious patients may have other rituals or practices
Offer organ donation, if potentially eligible	• Use standardized screening process for donation so no more patients are approached than necessary, but all potentially eligible patients are asked • MAID assessor/providers may not be knowledgeable about donation; education may be required • Organ donation organizations should develop toolkits and standard practices to assist MAID assessors, providers, and patients with these discussions
***Process theme 4: Death and aftercare***	
Support patient choices for location, route, and timing of death	• Provide clear information about feasible options regarding where, when, and how the MAID provision will occur • Provide the option of oral MAID provision to have further control only if available and feasible and provider is comfortable providing it • Explore with patients who they wish to have present during the provision; it may differ from family preferences
Choreograph the assisted death	• Accommodate patient and family requests when feasible, but be honest when some options cannot be done • Take exceptional care to be on time or early as delays in provision are very distressing to some patients and families • Care coordination between locations needs to be planned thoroughly and in advanced to ensure a smooth, confident provision process • Patients having control over their death is important to them • Dignity and independence through control brought by on MAID
Prepare the family for the patient’s death and follow-up	• Tailor education to the needs and understanding of the patient and family • Ask how much detail families want to know; some value specific information on syringes, colour change, time until heart stops while others may not • Support and brief the family before and after provision • Have a space for patients to gather afterwards without being rushed
***Transcendent theme 1: Coordination***	
Provide continuity of care	• Ask the patient which clinicians they would like involved in MAID • Engage the primary clinical team in the MAID process, including family physicians (if patient is in hospital) and sub specialists, irrespective of the patient’s location • Identify a “most responsible MAID clinician” or MAID coordinator
Provide education and manage expectations	• Provide multiple opportunities and methods for education, written and verbal • Check for understanding • Ensure that patient and family expectations are clear with respect to eligibility criteria, what assisted dying entails, and how flexible clinicians can be in providing assessments, assisting with preparation for death, provision, and aftercare
Facilitate access	• Patients may require advocacy from clinicians to overcome barriers to access • Check-in on tasks and next steps to ensure the process continues to move along
***Transcendent theme 2: Patient-centred care***	
Explore previous experiences with death and dying	• Probe for previous experienced with death and dying during assessments to help predict a patient and/or family’s needs throughout the process
Preserve dignity and privacy	• Provide compassionate care aimed at maintaining an individual’s dignity— what this means will vary between patients and families • Exercise even more than usual caution in keeping MAID information confidential than other personal health information
Be sensitive to stigma	• Be aware that patients and families may have experienced stigma and may take time to trust even well-meaning clinicians • Anticipate that patients and families will struggle with whom to share information about MAID request and how/when to disclose; support patients to tell their families and friends • HCPs should explore with patients and their family members how they are going to tell and how they are going and offer support for those conversations
Care for the whole family	• Engage families early and if not involved, explore the reasons why with the patient • Anticipate the complexity of emotions that family members will experience supporting their loved one’s decision for a hastened death • Bereavement and follow-up services should be provided to families, where available

*HCP* health care provider, *MAID* medical assistance in dying

### Themes related to MAID requests

i) *Promote MAID awareness*Participants described the need for efforts to raise awareness of MAID both in the general community and amongst clinicians. Patients required opportunities to be exposed to the possibility of MAID, as for many it was not clear if it was available, or how it could be accessed:

*I don’t think I saw any MAID information over at the cancer centre. I don’t think so and it should be there, it needs to be there. (Family member #812)*

ii) Explore requests for MAID. Patients requesting MAID described the need for clinicians to acknowledge and explore a patient’s wish to die, and to address their suffering. These early exploratory conversations could be a source of reassurance (if done well) or dissuasion (if done poorly or a clinician responded critically towards the MAID request).

*The initial patient contact when that decision is made is critical. I think the conversation that happened … helped him move his decision making forward.…it’s not about handing somebody a pamphlet and saying, “Here, here’s what it’s about.” (Family #512)*

iii) Facilitate written requests. Patients described struggles in identifying two independent witnesses for the written request, and those who relied upon friends and family were cognizant of the emotional burden this could put upon the witnesses.

iv) Move MAID requests forward quickly. Early delays were perceived to be due to the actions of the clinical team, due to either inefficiencies or even purposeful obstruction, highlighting the need for an efficient referral system for MAID requests:

*“Right. If my doctor didn't want me to do it, then he should have referred me to somebody else. He should have just not said, you're not eligible. And to my way of thinking he was saying, just keep on suffering. Because I was…(Patient #821)*

*“Because I think most situations, or at least in mine, drawing the process out may cause a lot of undue stress and frustration.” (Patient #421)*

### Themes related to MAID assessments

i) Assess in a safe, comfortable space and ii) Assessors need to build trust.

The clinical purpose of MAID assessments is to determine whether or not a patient meets eligibility criteria for an assisted death. The exploration of a patient’s suffering and their decision to pursue MAID, is a complex and sensitive activity. High quality assessments require trust of the clinician and comfort with the setting, whether it is an in person or virtual/telephone assessment:

*[The assessments] both happened at the hospital. There was a really nice quiet room and they were pleasant. It was a conversation.. It was like having dinner with somebody without the dinner and very calm, no pressure … (Family member #812)*

The assessments were cognitively and emotionally challenging; patients described feeling the pressure to perform for the assessors, to maximize their chance of being eligible.

*“I just wanted to give as much information as I could to persuade them that this was the right thing for me”(Patient #411)*

iii) Provide transparency around procedural safeguards. A family member or friend would sometimes be present during an assessment, described as a valuable experience for both patients and families as a support mechanism. Both parties appreciated the transparency around the purpose of the assessment, and the safeguards built into in the process:

*They kept asking her if she wanted to change her mind. And that's a good thing, because it always gives her the opening. And they asked us if we wanted her to change her mind. And we all basically said, no it's her decision whether she wants to go.*

*(Family #822)*

*“I liked that when I was present at the assessment and I was there to support my sister, I liked that I got sent out so they could interview her alone to make sure that she was not being unduly influenced” (Family member #412)*

iv) Integrate education into assessments. High-quality assessments resulted not only in a decision about eligibility for MAID, but also provided patients and families an opportunity to reflect upon options and ask questions of MAID clinicians:

*The second interview, you know, [provided] a bit more detail about how it would actually be handled on the day, the injections and that type of thing. So, more general information that just gave me more information and made me relax more. (Patient #411)*

### Themes related to preparation for dying

i) Support getting affairs in order. Patients and families made preparations for the patient’s death. While some of these were during Bill C-14’s legislated “waiting period” of 10 days between MAID request and provision of an assisted death, preparation for death sometimes began earlier, even preceding the patient’s written request for MAID.
ii) Monitor patient status. The issues raised by patients and families in preparation for dying were included monitoring patient status (as incapable patients were ineligible for MAID under Bill C-14), supporting reflection, and preparing for the patient’s death. Preparations included the need to get practical affairs in order, which in turn influenced patient perceptions of readiness for death:

*If your life is scheduled and you’re given a timeframe to work with, you do the estate planning and settlements. I think that type of planning makes death a whole lot easier. (Family member #712)*

iii) Support patient and family reflection. While awaiting the MAID death, patients and families reflected frequently on death and the decision for MAID, and the tradeoff between avoiding suffering and reduced life:

Investigator: *What makes the time between now and your death meaningful to you?* Participant: *Well, nothing. I wouldn’t say it was meaningful. The human instinct is to live as long as you can. It’s in there. You don’t know when it comes down to be your turn to die. As I said to my daughters even now, I’m dying to live but I’m not living to die.”(Patient #631)*

*You go up and down like an elevator, you know. Do I really understand this? Am I doing the right thing, and my family doing the right thing? Yeah, it's an elevator ride up and down, up and down. They just took all the fear away. I haven't been afraid of anything for the last couple months.....just a belief in what this program offers. I want it to get better but I like it the way it is so far. (Patient #911)*

These reflections were important for patients to decide if MAID was the right option for them, and for families to come to terms with the patient’s decision for a hastened death.

One major concern was that participants would lose capacity, and therefore become ineligible for MAID. Intertwined with this worry were decisions around symptom control, with patients and families sometimes deciding to withhold pain medications to avoid sedating effects and the development of delirium.

*She was happily surprised that she was further along to setting that date.....I think part of that was she was really worried she wouldn’t be competent to… You know, like it would be taken away from her. (Family #412)*

Some patients and families described the waiting period as agonizing:

*Oh I wish I was gone tomorrow…. But ten days. That’s a punishment. What for? I wasn’t that good a boy but that bad I wasn’t either. I’m lying here all day long waiting and waiting for the day to be over. And it’s a long, long day, you can believe me. When you wait for something. (Patient #521)*

iv) Incorporate spiritual, religious, and cultural death practices. The waiting period also presented an opportunity to integrate personal and family traditions, if wanted:

*They offered me spiritual advisors, social workers, whatever I wanted. And they would assist me to get in whatever I needed if I wanted and I basically turned them down. I felt my belief system, my internal setup was secure and at peace with the decision I was making so not an issue. (Patient #411)*

xxii) Offer organ donation, if eligible. Lastly, the waiting period also represented a chance to explore organ donation. While many patients would be ineligible due to their underlying disease, several described wanting to donate, if possible, as an act of giving:

*I think it’s a really good opportunity because most people, or a lot of people may feel that in choosing MAID it’s a very selfish thing to do. And this sort of helps balance that.(Patient #421)*

### Themes related to death and aftercare

i) Support patient choice for location, route, and timing of death. Patients and families valued having choice over many aspects of their death, including selecting persons who would be present. These decisions were often made in conjunction with the family and the care team:

*I think it’s enviable being the author of your own last chapter, as it were. Not everybody has that opportunity to make that decision. (Family member #912)*

ii) Choreograph the assisted death. An organized, professional approach to the actual day of death and preparing the patient and family for what to expect were described as important final steps in the patient’s care.

*He is the man for the job.....He knew exactly what he was doing and he knew exactly what to say and he knew exactly what to expect from people. We were totally happy and impressed with him. He just was totally on top of his game. He knew exactly, you know, what to say and do. (Family member #712)*

Coordinating the timing of events precisely was important, as even small changes in the scheduled time could be a source of anxiety.

iii) Prepare the family for the patient’s death and offer support afterwards. Family members valued education on what the patient’s death could look like, as many had not been present at the time of death of a loved one, or were unaware of how a MAID death would look and could feel. Others describe the value of clinicians providing support and information after the patient had died:

*He made a noise after the first one that sort of all surprised us....Just a snoring noise sort of....That was a surprise to all of us. We sort of weren’t sure what was happening. Maybe mentioning something like that, you know, could happen. Then you don’t feel so thrown off at that point. (Family member #232)*

#### Two transcendent themes: coordination and patient-centred care

Alongside these four “process” themes and sub-themes, two themes were developed across multiple steps of the MAID process. The first, *coordination* involved descriptions of how the complex MAID process was carried out in a smooth, professional, and predictable sequence. Central to this was continuity of care, as patients transitioned between sites, steps in the MAID process, or between health care teams. One of the central roles of the clinician or team coordinating MAID care was providing clear and consistent education and managing expectations. Patients and families were grateful when HCPs supported and facilitated access to MAID:

*I have to say, I never expected that level of kindness and cooperation, and information. It's like an information highway. You want to know something, we’ll tell you, just ask. .(1–1-009)*

The second theme which transcended all steps in the MAID process was *patient-centered care.* Central to this was exploring a patient and family’s previous experiences with death; individual needs and preferences, and restoring dignity by providing human connection and compassionate care:

*…we could laugh and joke and play music and, you know, have a little chat with all the team members and they were all absolutely amazing.... because the team was so compassionate and warm and friendly… (Family member #632)*

*I can see it in their eyes. It's not… It's not pity. It’s ‘how can we help you?’ What do we need to do to make it better. (Patient #911)*

Crucially, this also involved taking care of the whole family: patients described the importance of their families having a positive experience and support after the MAID death. Caring for the patient entailed caring for the family as well.

*Investigator: What was the most important thing that the healthcare team could do to help patients?*

*Participant: Look after my family. (Patient #131)*

Families acknowledged that supporting their loved one through the MAID process as a rewarding but emotionally complicated experience, and the need for counselling and bereavement services:

*I'm fully behind this whole program, I hope that it has continued success and that a lot more people are able to take advantage of it...Yeah, it would have been nice to have another couple of weeks, maybe a couple of months even, who knows, right but it wouldn't have been any fun for her and she'd already made up her mind that she did not want any part of that I respected that decision and I was at peace with the whole process (Family member #912)*

*I think that perhaps counselling could be a bigger part. I was lucky I had it, but I don’t know what you provide for MAID normally. I would think that counselling either for… also for family members. If my husband had say still been alive…I think he would have required counselling to… to face it. (Patient #141)*

Lastly, many participants recognized the possibility of stigma around MAID death, and how challenging it could be to discuss openly, with friends, family, or even the clinical team:

*I’ve had a conversation with one of my physicians here and, for him, ethically, religiously was a hard thing for him to do and he couldn’t condone it. But after talking with me, it has turned his head around to a better point of thinking that he would not want to endure this if it was this way. And all I can say is… try to put the shoe on the other foot. (Patient #811)*

## Discussion

We identified sixteen process themes and two transcendent themes related to patient and family perceptions of quality of care for MAID, along with a list of suggested practices, summarized in Table 2. While not supported by quantitative evidence, these suggestions are based upon the experiences of patients and family members who have been through the MAID process. Health care clinicians and organizations in Canada may use these to guide practice, while researchers may use them to inform research. For international readers, these results can still be applied to practice (if legislative parallels exist). These results can also assist readers in jurisdictions where MAID is not legal to understand the perspectives and needs of patients who request a hastened death, and perhaps to alleviate some aspects of their suffering.

### What this study adds

In addition to the practice suggestions we provided, the two transcendent themes—coordination and patient-centred care— are particularly noteworthy, as these speak broadly to the opportunities and challenges posed by MAID as a therapeutic intervention. The complex medico-legal nature of MAID requires considerable organization on the part of patients, families, and health care teams. This coordination straddles traditional health care silos such as community vs. hospital settings, or specific clinical programs. MAID is a novel clinical practice which requires dedicated organization to ensure smooth transitions in care, completion of paperwork and reporting, coordination of the numerous decisions leading to the patient’s death. None of these activities clearly fall within a traditional clinical role. While some individual clinicians may function without dedicated MAID coordination services —either because their practice is dedicated to MAID or case volumes are very small—most are likely to benefit from the formalization of the role. Study participants’ responses implied that a lack of coordination in the MAID process could be hugely disruptive, potentially resulting in prolonged suffering or loss of eligibility for MAID. Poorly coordinated care is unlikely to lead to a satisfying experience for any participant in MAID— the patient, the family, or the clinical team. The widespread formation of MAID care coordination services and the MAID coordinator role are thus likely to be a permanent, rather than a transitional aspect of MAID in many settings [15].

We identified a complex relationship between patients, families, and the health care team which suggests a need to recalibrate what “patient-centred” means in the context of assisted dying. Patients who request MAID are often concerned with the impact of their choice upon others, especially their family and friends. Decision-making around assisted dying is often framed in terms of either the physician-patient dyad or patient-family dyad [16]; by contrast we noted in our study that decision-making was more often a triad, involving all three of patient, family, and clinician. In striving for the best possible death experience, participants described a need to compromise— patients suffer longer to allow families to prepare for their death; families support their loved one’s choice to die sooner; and clinicians bend policy to provide compassionate care to patients and families. The complex challenge of providing “patient-centred” care lies in balancing these conflicting values, needs, and preferences [17]. While patient-centred care is held as an ideal, reality may be a more complicated compromise between all three parties in MAID decisions. Fostering an honest discussion of values with *all* participants, rather than simply following on the patient’s stated choices, is essential to creating a patient-centred MAID experience, even if it is ultimately the patients capable choices which clinicians strive to uphold [18].

A systematic review by Gamondi et al. summarized a number of studies describing the cognitive and emotional responses of family members to a loved one’s assisted death, and be unique aspects of bereavement and aftercare in this context [4]. However, there are relatively few studies describing the perspectives of patients and families on procedural aspects of care, possibly due to the heterogenous medicolegal contexts of assisted dying around the world. Comparisons with other studies suggests there is some transferability of this study’s findings, especially in Canada. Brown et al. conducted a qualitative study including 5 patients, 11 family members, and 14 health care providers in Saskatchewan, identifying sustainability, pathway ambiguity, lack of support for care choices, institutional conscientious objection, navigating care, post-death documentation, and legal stipulations as being primary procedural concerns of patients and families [19]. This study reaffirms that ambiguity around care processes and struggles coordinating care are major challenges faced by patients and families. A separate report of the same interviews explored considerations for providing patient and family centred care which are also align with those in the this study [20]. The present study builds upon this work by integrating both procedural and patient-centred aspects of MAID, and providing concrete practice suggestions.

This study’s results also parallel those of Hales et al.*,* who conducted a multi-methods study including 11 family members [21]. They identified six areas for improvement, 3 organizational (process clarity, scheduling challenges, 10-day reflection period) and 3 experiential (clinician judgement/objection, secrecy/privacy, and bereavement support). These correspond closely to several themes we identified. While family bereavement and counselling were mentioned, they were not as prominent in this study, possibly because the interviews with families occurred fairly quickly after the patient’s death and interviews focused on providing support immediately around the patient’s death, rather than long-term bereavement support (6–8 weeks). Participants perspectives of MAID may change with time after the death of a loved one, with bereavement playing a larger role in perceptions of quality after more time has passed [22]. Future research will be useful to determine which long-term supports will be most useful for families [23–25].

The coherence of results between this study, and others conducted in other Canadian centres suggests that the experiences and expectations of Canadian MAID patients and their family members may be similar in a variety of practice settings, with some caveats (described below). The results of this study will be used to develop patient and family satisfaction surveys, which will undergo testing at multiple Canadian centres and thus test this hypothesis. If the surveys demonstrate adequately robust psychometric properties, they will be useful in assisting clinicians and institutions in MAID research and quality improvement. Other important research topics can include testing communication strategies, screening tools for patients who may be interested in MAID, and bereavement supports for families.

### Strengths and weaknesses of the study

This study has several strengths, including its multi-centre recruitment strategy, which aimed to recruit a broad but representative sample of both MAID patients and their families. This is supported by the participant demographics, which are consistent with those reported by the Canadian Federal government, suggesting we interviewed a reasonably broad sample of Canadian MAID patients and families according to these criteria [1]. As well, by recruiting patients and families, we were able to identify practical areas to improve the MAID process from the perspective of both groups. Weaknesses of this study include the large proportion of respondents from Ontario, lack of representation from rural and remote communities, lack of data collection on patient and family race and ethnicity, and inability to account for the impact of centres’ different practice models, care teams, and patient populations. These results may thus not be transferrable within specific socio-demographic contexts. Other weaknesses include the use of telephone/video for some interviews, which may have enhanced recruitment but also have had an impact upon participant contributions to the study. As well, we did not conduct member-checking with families to ensure our interpretations were consistent and resonant with their experiences, although bereaved relatives in MAID may have been willing to participate in this and long-term follow-up, as they are in other palliative care research [26]. Lastly, while we aimed for a low-inference, qualitative descriptive approach to our data, there is a significant interpretive aspect to our use of thematic analysis as we applied it to the study objective, and some would argue that content analysis would be a preferred analytic approach [27].

## Conclusion

Patients and families described a variety of successes and challenges in the provision of high quality care during the MAID process. We identified numerous opportunities to enhance the experiences of those involved in a patient’s assisted death.

## Supplementary Information

**Additional file 1.**

**Additional file 2.**

## Data Availability

The datasets used and/or analysed during the current study available from the corresponding author on reasonable request.
