# Supplementary material for: How can we improve the experiences of patients and families who request medical assistance in dying? A multi-centre qualitative study
Source: BMC Palliat Care. 2021 Dec 8;20:185. doi: 10.1186/s12904-021-00882-4 (PMC8653618; doi:10.1186/s12904-021-00882-4)
Supplement: Supplementary file 1 — Additional file 1. [file 12904_2021_882_MOESM1_ESM.docx]

**Supplementary material 1: Study Interview Guides**

Simon Oczkowski, Diane Crawshaw, Peggy Austin, Donald Versluis, Gaelen Kalles-Chan, Michael Kekewich, Dorothyann Curran, Paul Miller, Michaela Kelly, Ellen Wiebe, Andrea Frolic

*How can we improve the experiences of patients and families who request medical assistance in dying? A multi-centre qualitative study*

**Patient Interview Guide**

I will record the interview and write some notes as we go. This interview is confidential, and no data that could identify you will appear in the final report. We can skip questions that you prefer not to answer, and if you like, we can stop the interview completely at any point. We can also break up the interview it completing it all at once is too difficult for you.

Before we start, do you have any questions?

(Answer any questions they may have about the study rationale, methods, or purpose)

**1. Our first set of questions will be about when you requested MAID. Not just when you started to think about it, but how you went about asking your health care team about it, and how you completed the written forms.**

a. What made you want to seek medical assistance in dying, as opposed to other end of life options?

b. What were the steps you took when requesting MAID?

c. What made requesting MAID easier for you?

d. What made requesting MAID more difficult for you?

e. What, to you, is the most important thing that the health care team can do to help a patient requests MAID?

f. If you could change something about the process of requesting MAID, what would it be?

**2. After you requested MAID, you have had to undergo assessments to see if you are eligible for assistance in dying under Canadian law. In these next questions we will ask you about what it was like during those assessments.**

a. Could you describe how and when those assessments happened? (Probes: who; where; when)

b. What made the assessments easier for you?

c. What made the assessments more difficult for you?

d. Did the assessment process help you understand your condition, care options, or dying process any differently?

e. Were you asked about your preferences for organ donation, if so, what did you think about that? If not, do you wish you had been?

f. What, to you, are the most important things for a health care team to do to help a patient who is being assessed for MAID?

g. If you could change something about the assessment process, what would it be?

**3. According to Canadian law, there is a mandatory 10 day reflection period between when a patient requests MAID and when assistance in dying is provided. This is so patients have an opportunity to think about their request. Some patients request assistance in dying as soon as the 10 day period is completed, other patients wait longer. These next questions will ask about this waiting period.**

1. What have you thought about during this reflection period?
2. What makes the time between now and your death meaningful to you, if it is meaningful at all?
3. What have you done to prepare between now and when you die?
4. What else do you need to prepare between now and when you die?
5. What could the health care team do to help you during the waiting period?
6. If you could change something about this waiting period, what would it be?

**4. Some patients who request MAID die with medical assistance, while others choose not to proceed, or get sick too quickly to die with medical assistance. This next set of questions will ask you to think ahead, to the time of your death, whether or not you eventually die with medical assistance.**

1. What would your ideal death look like? (Probes: Who, where, when)
2. What to you is the most important to ensure a good death, and why do you think that? (Probes: speed; painless; control; previous experiences with death)
3. How important is it to have control over your death? (Probes: choose the day of death; choose the time of death; control the actual administration of medications)
4. What are you most worried about when you die?
5. Are religion or spirituality important in helping you prepare for death, and if so how?
6. You must have capacity for decision-making (be of “sound mind”) right up until the time of MAID. Are you worried you may not meet that requirement? (Probes: if ‘concerned’, ask if they have done anything to make sure they remain of sound mind/capable)
7. What would you think should be done if you can no longer receive MAID?

**5. We are just about done the interview. I would just like to ask a few questions about your experiences overall.**

1. What would ‘quality’ care in assisted dying look like to you?
2. What needs to be done to ensure that quality care is provided to patients who request MAID?
3. Before we finish, is there anything else you would like to say?

*Ensure that all information needed to fill out the patient’s preferences (#7-9) on the demographic form has been collected during the course of the interview. If it has not, then ask the following questions to ensure that the information is collected.*

1. *Very briefly, have you thought about your preferred location for assisted dying? (home, hospice, hospital, other….) or do you not have a preference?*
2. *Very briefly, have you thought about your preferred route for the medications in assisted by dying? (oral - by mouth, or intravenous - by injection), or do you not have a preference?*
3. *Very briefly, have you thought about your preferred timing for assistance in dying? (within the week; one week to one month; greater than one month; or do you not have a preference?*

Thank you so much for taking the time to speak with me.

**Family Member Interview Guide**

Thank you so much for speaking with me today and participating in this study.

In this study, we are trying to understand the values, needs, and preferences people have for medical assistance in dying. To do this, we are interviewing patients who are eligible for assisted dying, their families, and their health care providers (the physician or nurse who is assessing or providing MAID). We want to know what is really important to people so that in the future, ensure that the quality of care we provide to patients who request assisted dying is as good as it can be. As a family member who has been involved in the MAID process, we believe it is important to take into account your experiences.

I will record the interview and write some notes as we go. This interview is confidential, and no data that could identify you will appear in the final report. We can skip questions that you prefer not to answer, and we can stop the interview completely if you like at any time.

**IF PATIENT HAS DECLINED/IS UNABLE TO BE INTERVIEWED, ALSO ASK QUESTIONS IN BOLD.**

Before we start, do you have any questions?

(Answer any questions they may have about the study rationale, methods, or purpose)

**1. Our first set of questions will be about when X requested MAID. Not just when X started to think about it, but how X asked their health care team about it, and completed the written forms.**

a. What made X want to seek medical assistance in dying, as opposed to other end of life options?

b. What were the steps X took when requesting MAID?

c. What do you think made X’s request for MAID easier for you? **(FOR X?)**

d. What do you think made X's request for MAID more difficult for you? **(FOR X?)**

e. What do you think is the most important thing the health care team can do to help a patient who requests MAID, and their family?

f. If you could change something about the process of requesting MAID, what would it be?

**2. After X requested MAID, X had to be assessed by two health care providers to see if they were eligible for assisted dying under Canadian law. In these next questions we will ask you about what it was like during those assessments.**

a. Could you describe how and when those assessments happened? (Probes: who; where; when)

b. What do you think made the assessments easier for you? **(FOR X?)**

c. What do you think made the assessments more difficult for you? X? **(FOR X?)**

d. What are the most important things for the health care team can do to help a patient who is being assessed for MAID, and their family?

e. If you could change something about the assessment process, what would it be?

**3. According to Canadian law, there is a mandatory 10 day reflection period between when a patient requests MAID and when assistance in dying is provided. This is so patients have an opportunity to think about their request. Some patients request assistance in dying as soon as the 10 day period is completed, other patients wait longer. These next questions will ask about this waiting period.**

1. What did you and X talk about during the reflection period?
2. What did you **(DID X)** do to prepare for X’s death during the reflection period?
3. What could the health care team have done to help you and X during the waiting period?
4. If you could change something about the waiting period, what would it be?

**4. Some patients who request MAID die with medical assistance, others are chose not to proceed, or get sick too quickly to die with medical assistance. This next set of questions will ask about the actual time of death, with or with out assisted dying.**

1. What happened when X died? (Probes: Who, where, when)
2. What do you think X would have needed to have the best death possible?
3. What to you **(TO X?)** is the most important ensure a good death?
4. How important was it for X to have control over his/her death? (Probes: choose the day of death; choose the time of death; control the actual administration of medications)
5. What were you most worried about before X died? **(WHAT DO YOU THINK X WAS MOST WORRIED ABOUT BEFORE HE/SHE DIED?)**
6. Were religion or spirituality important in helping you prepare for X’s death? **(Were religion or spirituality important to X in preparing for their death?)**
7. A patient must have capacity for decision-making (be of “sound mind”) right up until the time of MAID. Were you worried X may not meet that requirement? (Probes: did you or X do anything to try and make sure X would be of sound mind on the day?)
8. What would you think should have been done if X could no longer receive MAID?

**5. We are just about done the interview. I would just like to ask a few questions about your experiences overall.**

1. What would ‘quality’ care in assisted dying look like to you?
2. What needs to be done to ensure that quality care is provided to patients who request MAID?
3. What do you think needs to be done to ensure that family members have a quality experience when family are receiving aid in dying?
4. Before we finish, is there anything else you would like to say?

Thank you so much for taking the time to speak with me.

**Health Care Provider Interview Guide**

Thank you so much for speaking with me today and participating in this study.

In this study, we are trying to understand the values, needs, and preferences people have for medical assistance in dying. To do this, we are interviewing patients who are eligible for assisted dying, their families, and their health care providers (the physician or nurse who is assessing or providing MAID). We want to know what is really important to people so that in the future, we can ensure that the quality of care provided to patients who request assisted dying is as good as it can be. As a health care provider who is involved in MAID, we believe it is important to take into account your experiences.

I will record the interview and write some notes as we go. This interview is confidential, and no data that could identify you will appear in the final report. We can skip questions that you prefer not to answer, and we can stop the interview completely if you like at any point.

Before we start, do you have any questions?

(Answer any questions they may have about the study rationale, methods, or purpose)

**1. Our first set of questions will be about X’s request for MAID.**

a. What made X want to seek medical assistance in dying, as opposed to other end of life options?

b. What were the steps X took when requesting MAID?

c. What made X’s request for MAID easier for you?

d. What made X’s request MAID more difficult for you?

e. What do you think is the most important thing a care provider can do to help a patient who requests MAID?

f. If you could change something about the process of requesting MAID, what would it be?

**2. After X requested MAID, you assessed him/her to determine eligibility for MAID under Canadian law. In these next questions we will ask you about your experience during your assessment.**

1. Could you describe how the assessment happened?

b. What do you think made the assessment easier for you?

c. What do you think made the assessment more difficult for you?

d. What makes a good quality assessment for MAID?

e. If you could change something about the assessment process, what would it be?

**3. According to Canadian law, there is a mandatory 10 day reflection period between when a patient requests MAID and when assistance in dying is provided. These next questions will ask about this waiting period.**

1. What do you think were the most important things for X to do during the waiting period?
2. What could the health care team have done to help X during the waiting period?
3. What were your biggest concerns during the waiting period?
4. If you could change something about the waiting period, what would it be?

**4. Some patients who request MAID die with medical assistance, others chose not to proceed, or get sick too quickly to die with medical assistance. This next set of questions will ask about the actual time of death, with or with out assisted dying.**

1. What happened when X died?
2. What do you think X’s ideal death would have looked like? (Probes: Who, where, when)
3. What do you think X needed to have a good death?
4. What role, if any, did religion or spirituality play in X’s death?
5. What do you think the health care team could do to ensure patients requesting MAID the best possible end of life experience?
6. What do you think the health care team could do to ensure families have the best possible end of life experience?
7. What is most important to you, as a health care provider, when a patient receives an assisted death?
8. A patient must have capacity for decision-making right up until the time of MAID provision. Were you worried X may not meet that requirement?
9. What would you think should have been done if X could no longer receive MAID?
10. What was your biggest concern about X receiving medical assistance in dying?

**5. We are just about done the interview. I would just like to ask a few questions about your experiences overall.**

1. What would ‘quality’ care in assisted dying look like to you?
2. What needs to be done to ensure that quality care is provided to patients who request MAID?
3. As an assessor/provider, what do you need to have a quality experience when providing MAID? (Probes: number of cases/year; debriefing/supports)
4. Before we finish, is there anything else you would like to say?

Thank you so much for taking the time to speak with me.
