# Supplementary material for: How can we improve the experiences of patients and families who request medical assistance in dying? A multi-centre qualitative study
Source: BMC Palliat Care. 2021 Dec 8;20:185. doi: 10.1186/s12904-021-00882-4 (PMC8653618; doi:10.1186/s12904-021-00882-4)
Supplement: Supplementary file 2 — Additional file 2. [file 12904_2021_882_MOESM2_ESM.docx]

**Supplementary material 2: Expanded table of themes, sub-themes, and exemplar quotations**

Simon Oczkowski, Diane Crawshaw, Peggy Austin, Donald Versluis, Gaelen Kalles-Chan, Michael Kekewich, Dorothyann Curran, Paul Miller, Michaela Kelly, Ellen Wiebe, Andrea Frolic

*How can we improve the experiences of patients and families who request medical assistance in dying? A multi-centre qualitative study*

| **Theme/item** | **Exemplar quote (s)** | **Synthesis of findings and suggestions** |
| --- | --- | --- |
| **Process themes 1: MAID Requests** | | |
| **Promote MAID awareness** | “it was a social worker on the floor she was talking to me about palliative care and brought up the fact that there was this thing called MAiD. I had no idea at this point that assisted dying was a possibility in Ontario at this point. So this was like a burst of sunshine, a ray of sunshine to me that this option was available.” (Patient #411)  “I don’t think I saw any MAiD information over at the cancer centre. I don’t think so and it should be there, it needs to be there.” (Family member #812)  “Let’s educate society and then it becomes a comfort instead of a resistance” (Family member #612)  *Investigator:* So what to you is the most important thing that the healthcare team could do to help a patient in requesting MAiD? *Participant:* I think probably more education for GPs.” (Patient #141) | - Public awareness of MAID may not be as common as HCPs perceive - Patients and families depend upon HCPs to understand prognosis and treatment options (including MAID) - The care team, especially social workers, are an invaluable source of information for patients and families about MAID   ***Practice suggestions***   - Hospitals and clinics which care for patients at high risk of death should have information about MAID publicly available so patients are aware of this option - Awareness efforts should target the general public and families (not just patients) to help prepare them for the possibility their loved ones may choose this options - Awareness and educational material can take many forms; posters, pamphlets, videos - Institutions should have clear policies around HCP bringing up MAID as an option, consistent with CAMAP guidance |
| **Explore requests for MAID** | “Yeah. not just be listened to but acknowledged. I think that’s really important.”  (Family member #812)  *Investigator:* What would quality care in assisted dying look like to you?  *Participant:* Exactly what happened with my dad. I would say that the initial patient contact when that decision is made is critical. I think the conversation that happened when I informed Dr. [NAME] that’s what he wanted to do helped him move his decision making forward. I’m not saying it has to be Dr. [NAME]. I’m just saying, you know, that first medical touch point. It’s not about handing somebody a pamphlet and say, here, here’s what it’s about  *Investigator:* It’s about that conversation.  *Participant:* That’s right. She never did that. She gave it to me. Which is fine. But she never gave it to my dad. And I think that the fact that she talked to him about it. In real life… it was healthier for him to process that decision“(Family member #512) | - Patients requests for MAID may be indirect and are not always explored by HCPs - Exploring other options alongside MAID can help patients navigate care choices - Patients and families do not always know when a formal MAID process has been “started"   ***Practice suggestions***   - HCPs should be aware that patients may struggle to bring up MAID, even if they are seriously considering it - Develop toolkit/communication strategies to assist HCPs in how to recognize and explore possible MAID requests - Use direct language to acknowledge and explore a patient’s request for MAID |
| **Facilitate written requests** | “The forms could be made simpler. Everything for the one person on one page and… instead of, you know, turning pages and having to have oh, I forgot, this should go here, you know. “ (Patient #711)  “…the witness section is something that… I understand you have to ask a witness, but asking a witness for some people that you know to be witness of my dying… My sister is my closest friend and sister and family and all that and she’s the one who did it, but I feel bad for her.” (Patient #231)  “You know, I think the time that it took too, because I was… You know once he had made the decision… And not being able to tell anybody I had to kind of… I was trying to find two witnesses for his signature. You know, I live in Nova Scotia, so I really didn’t know a lot of people. I was trying to find two, you know, witnesses who we didn’t know and Dr [NAME] said, oh sometimes there’s people who, you know, are going through the same thing here that will sign and it was just that… I found that to be difficult. Um, you know, I had asked a couple friends of mine who um, you know, based on religious beliefs, weren’t comfortable with that, which was fine. “ (Family member #512) | - For many patients, the need to find two independent witnesses is a major obstacle in the MAID process - Patients can feel uncomfortable asking friends/family who are eligible under the law, perceiving this as burdensome - The paperwork used for written requests is often complicated and difficult to complete correctly   ***Practice suggestions***   - Written requests, and other patient/family-facing paperwork should be as simple and accessible as possible - Provide assistance with completing the written requests and witnessing, to avoid the need for corrections/revisions - Provide contact information for Dying with Dignity which can provide volunteer witnesses, or have institutional volunteers who can assist inpatients |
| **Move MAID requests forward quickly** | “Because I think most situations, or at least in mine, drawing the process out may cause a lot of undue stress and frustration.” (Patient #421)  “Right. If my doctor didn't want me to do it, then he should have referred me to somebody else. He should have just not said, you're not eligible. And to my way of thinking he was saying, just keep on suffering. Because I was (Patient #821)  “I don’t want to suffocate, I don’t want to go up in flames, and I don’t want it to drag on. Starting to wonder about that now because everywhere I see it, it has been dragging on contrary to practitioners’ statements” (Patient #331)  “But she asked her, she said, I don't want to be here anymore. And she said, well, we can help you with that. And she said, go see the doctor and then if not, she could help her do it. But we figured go through the doctors, right. That's what he said. He said, ‘no never had it on file.’ And that is a lie. I will swear to God, that's a lie. Anyway, so we had to wait for you guys to step in.” (Family member #622)  “Because everybody’s got their own little silo that they live in, but to say that we have this… this opportunity in Canada to do this. We have MAiD. And it’s good, but we also have to follow the same policies, procedures, and we can’t… I don’t think you really can discriminate anymore. We just… We can’t.” (Family member #812) | - Patients often have to request MAID several times before a HCP takes steps to move the process forward; some HCPs seem to delay the process, causing patient distress - Patients and families are often uncertain of who is accountable for moving a MAID request forward - Delays and inconsistent messaging from HCPs are a source of frustration and distress for patients and families, who expect it to be a smooth, well-integrated process   ***Practice suggestions***   - Have clear institutional policy/protocol for referring patients who request MAID to HCPs who will take responsibility for the process - Policies should be supportive of conscientious objectors but mandate rapid refer/transfer patients so as not impede timely response to MAID requests - Provide written information to patients and families mapping out the MAID process so they can keep track of next steps and hold the system for moving the request forward |
| **Process themes 2: MAID Assessments** | | |
| **Assess in a safe, comfortable space** | “They both happened at the hospital. There was a really nice quiet room and they were pleasant. It was a conversation….. It was like having dinner with somebody without the dinner and very calm, no pressure and they kept insisting, even if you do… are approved for MAiD, you don’t have to. Nothing says you have to. So that was reinforced. (Family member #812)  “that assessment took place via Skype. It lasted about an hour and a half and was an excellent… You know, she had in front of her, of course, my CT scans, my bone density, my entire medical history all of the medications I’ve been on for years all of the broken bones etc.” (Patient #141) | - Patients and families appreciate flexible, timely assessment processes - Patients and families are often anxious about assessments, viewing them as a “test” for which they must “perform" - Private, quiet environments put patients and families at ease and allow for open dialogue   ***Practice suggestions***   - HCPs should acknowledge the stress the patient is under, and that the assessment is not a test, but a conversation; offer to explain the legal criteria for MAID - Provide an outline of the purpose and content of the assessment - Use a conversational manner rather than “checklist” approach to encourage information sharing - Identify private spaces for assessments; consider use of videoconferencing/telemedicine |
| **Assessors need to build trust** | “What made it difficult was repeating the same thing all the time and you want to make sure you don’t forget something. I guess if you’re asking for it and you forget something…. that could have brought the yes to proceed with MAiD if that… I was very nervous for that and I just wanted to make sure I was saying the same thing what I did on the first one to the second one and that’s all the truth” (Patient #231)  “That could have been the first one in the place of my G.P. a more unbiased opinion, but at the same time my G.P. knows what I’m like. So she has valuable input” (Patient #531)  “I just wanted to give as much information as I could to persuade them that this was the right thing for me”(Patient #411)  “See they just walk in the door and they sit down and you talk. Nobody tells you who they are or why exactly they're there.” (Patient #911) | - Patients and families find assessments to be stressful; they often lack detailed recall of exact details of who was present - When assessments are done by HCPs without a pre-existing relationship to the patient (eg. a MAID team member), patients and families may be distrustful   ***Practice suggestions***   - HCPs need to introduce themselves and their role in the MAID process and the patient’s care - Include, where possible, HCPs the patient/family know (eg. family physician, hospital most responsible physician) in the assessment process |
| **Provide transparent procedural safeguards** | “They kept asking her if she wanted to change her mind. And that's a good thing, because it always gives her the opening. And they asked us if we wanted her to change her mind. And we all basically said, no it's her decision whether she wants to go.”  (Family member #822)  “Before that they asked my family to leave so that they could talk to me directly and made sure I wasn’t being coerced and that was safety point that I loved.” (Patient #411)  “I liked that when I was present at the assessment and I was there to support my sister, I liked that I got sent out so they could interview her alone to make sure that she was not being unduly influenced” (Family member #412) | - Sometimes onerous, many patients and families appreciate the value of the two assessment as a safeguard against coercion   ***Practice suggestions***   - Reinforce during the assessments that the patient can change their mind or stop the process at any time - Having a portions of the assessment with and without family present is recognized as a safeguard - Having two HCPs present for each assessment (eg MD/NP with an allied health member) can be a safeguard for both the patient and HCP |
| **Provide education** | “So obviously it was really good for both of us to hear her say that and knowing it was her own wishes.... It creates a neutral place to have that conversation because it’s a difficult conversation sometimes for, you know, a person to have with their family. Like when do you raise it? People are coming to visit you in hospital, when is the right time to talk about what my choices are for my dying?” (Family member #412)  “During the questioning of that first interview, as I expanded my answers it became clearer to me that this was what I wanted. The questions were making me give information about why I was making choices, why this would work or, you know, what inspired me. And everything was just making it more solid within my decision making. The second interview, you know, didn’t really need all that but a bit more detail about how it would actually be handled on the day, the injections and that type of thing. So, more general information that just gave me more information and made me relax more. It’s all in hand...... And I think I would highly recommend against getting rid of that. You need that second interview.” (Patient #411) | - Patients view assessments as an opportunity is to express their feelings and solidify their desire for MAID - Assessments can help families understand their loved one's motivations for requesting MAID - Patients and families want the unvarnished information about the process   ***Practice suggestions***   - Emphasize that the assessment is also an opportunity for patients to learn about MAID, and that during the assessment they may change their mind or solidify their desire - If the patient is willing, encourage close family members to be present during the assessment, so they can understand the patient's motivations for MAID - Use the assessment as an opportunity to educate and prepare patients and families; MAID assessors and providers often have the best information on what to expect and prepare for |
| **Process themes 3: Preparation for dying** | | |
| **Support getting affairs in order** | “If there could be a guide of sorts, like a very basic booklet on legal issues that you might encounter and maybe a separate booklet on societal issues.” (Family member #122)  “If your life is scheduled and you’re given a timeframe to work with, you do the estate planning and settlements I think that type of planning makes death a whole lot easier.” (Family member #712)  “We said, if she says no now she's going to be screwed because she's got no clothes no place to live, no furniture.” (Family member #822)  “Because it was like, ding that’s done, ding that’s done. So he had his mental check list so you could just see as things started to get completed he became very peaceful, which was good.”  (Family member #812)  “He wanted to make sure that everything was in order. Like all his paperwork and taking care of his kids....He took that time to if you have any sort of hard patches with, you know, your siblings or whatever. You just, you know, wanted to make sure you settle it. You know, and everything was taken care of.” (Family member #232) | - While some patients and families were well prepared, some patients’ clinical conditions change quickly with little advance time for getting affairs in order - Family and friends, rather than patients, often made arrangements for personal finances, funeral arrangements etc. - Practical arrangements are often perceived as a way of finding closure to life - Some patients used the reflection period as an opportunity to close and settle relationships   ***Practice suggestions***   - Develop a list of tasks to be completed, including estate planning - If possible, involve social workers to assist in these decisions and processes as some families may find making arrangements cathartic while for others is may be burdensome |
| **Support patient and family reflection** | Constantly about is it the right thing for my family. And do they understand it’s what I want and it’s sort of… with the idea that go smiling when you go out, but going out with a smile because it’s what we want and it is what I want and I hope it’s what they want and I go out, again, cognitively and being able to talk to each other and understand each other just hope they are on the same page. I know it’s a hard way to go and all emotional suffering and that.  But just that they understand and they’re onboard with it....Good team here. Good compassion. Taking care of the family while taking care of me.” (Patient #221)  “You go up and down like an elevator, you know, do I… do I really understand this, am I doing the right thing, and my family doing the right thing. Yeah, it's an elevator ride up and down, up and down. They just took all the fear away. I haven't been afraid of anything for the last couple months..... just a belief in what this program offers. I want it to get better but I like it the way it is so far.” (Patient #911)  *Investigator:* What did you do to prepare for [NAME] death during that period of time that you were waiting?  *Participant:* Got rid of all his clothes. Got rid of most of his clothes. Like he said, I’m supposed to be dead before you start that. So no preparation otherwise. We just lived the day. That sounds terrible doesn’t it? Got rid of all his clothes...No, we kept a couple of pairs of shorts back just in case we had a heat wave, you know. (Family member #632)  *Investigator:* What makes the time between now and your death meaningful to you? *Participant:* Well, nothing. I wouldn’t say it was meaningful. The human instinct is to live as long as you can. It’s in there. You don’t know when it comes down to be your turn to die. As I said to my daughters even now, I’m dying to live but I’m not living to die. (Patient #631)  “She controlled her life up until the end. She wanted to make sure that she talked to as many people in the family as possible that, you know, and gave them time to, you know, listen to her and hear her thoughts and ideas so that they could, you know, understand the reason for making her decision.... she wasn’t leaving family so much as she was, you know, leaving the illness.” (Patient #721)  “As soon as I put my signature on a document, it’s done. The decision is made” (Patient #631) | - Patients and families may view the 10 days of the reflection period differently; for some it is an opportunity to savour the last days of life, for others it is burdensome prolonging of a death - Patients and families appreciate small gestures which make them feel specially cared for in the days leading up to the provision   ***Practice suggestions***   - Prepare patients and families for the emotional nature of the reflection period by acknowledging that complexity of emotions - Ask and explore what families and patients need to make the most out of the reflection period; the person best able to do this will vary depending on wishes and preferences - Specialist palliative care consultation, if not already done, can provide valuable support |
| **Monitor patient status** | *So the waiting period how did that go?…* “It was shitty. I mean at least at that point I could tell people and I was able to put it into perspective....I found that to be the hardest part was just the time from when he decided to when it actually happened was a long period of time.” (Family member #512)  “I still think that people should be able to video while they are able, video their request of what they need and I think that that should be the thing that should be accepted. This was the choice and the illness has taken him to this point and he can’t say it. I think that would be a fair way to do things for people. I know the law would have to change for that though.” (Family member #632)  “She was happily surprised that she was further along to setting that date.....I think part of that was she was really worried she wouldn’t be competent to… You know, like it would be taken away from her. (Family member #412)  “I think ten days waiting. It’s a good thing. People who are going through this process probably want to get to that point. You know, it’s getting closer so they’ve probably got a date in their head already in a lot of cases might say as soon as that ten days is over they’ve already planned to go that week, whereas we were just on hold. You know, depending on how [NAME] went along. So for me nothing needs to change” (Family member #432)  Make it three days because ten days may how many. They’ve been lining up these people here. You know it’s… I know it’s a new project, but it needs work I guess. It’s much too long, ten days. Much too long. The suffering for the poor bastard and very much so… and oh I wish I was gone tomorrow…. But ten days. That’s a punishment. What for. I wasn’t that good a boy but that bad I wasn’t either. I’m lying here all day long waiting and waiting for the day to be over. And it’s a long, long day, you can believe me. When you wait for something” (Patient #521)  *What do you think should have been done if [NAME] in the end, wasn’t of sound mind?* “I think… It needs to proceed because he was of sound mind. It’s like a will. He was of sound mind when he did that.” (Family member #812) | - Time can feel distorted to patients and families during the reflection period; very long and or very quick - The logistics about 10 day reflection period and possible shortening of the reflection period was sometimes confusing to patients and families - For many patients and families, the reflection period is centred around worries about the loss of capacity to consent - Patients will sometimes forego pain meds in order to maintain capacity; MAID may be perceived as more desirable than the relief of physical suffering - Largely there was support for MAID continuing if an eligible patient’s capacity was lost during the reflection period   ***Practice suggestions***   - Identify patients at risk of losing capacity early, and ask patients/families if this is a source of anxiety for them - Explain available options in the event that capacity is lost - For patients judge to be a high risk of losing capacity, consider close monitoring to allow for rapid reassessment and potential shortening of reflection period if capacity loss is probable - Offer to reassess patients who have lost capacity as this may fluctuate - Discuss with patients palliative care options which may best preserve capacity, if MAID is their overriding preference |
| **Incorporate patients’ spiritual and cultural death practices** | “And she was also going to manage to have someone come in and say a couple of prayers” (Patient #821)  “A little spooky but comforting. My uncle also said the same thing. He said I already know that these people are lined up waiting for him and that this is what’s going to happen and so for from that respect um, you know, there was spirituality that wasn’t my own but that became… not heavily relied on, but a comfort.” (Family member #512)  “They offered me spiritual advisors, um social workers, whatever I wanted. And they would assist me to get in whatever I needed if I wanted and I basically turned them down. I felt my belief system, my internal setup was secure and at peace with the decision I was making so not an issue. But the offers were made there definitely, you know, have you met our priest… our Chaplin. Oh yes, I’ve had long discussions with her actually. (laughter) But that was on a previous visit. But I didn’t think I would… I really had anything to gain from it.” (Patient #411)  “Well, God is usually in charge of our date.....It’s like planning a c-section for a baby. This is when it’s going to be and the time. You’re anticipating a date for somebody to die. It’s kind of a weird, weird thing” (Family member #212) | - Spiritual and religious beliefs and preferences vary widely from patient to patient, and within patients/families - Patients and families may be uncertain of how their faith communities would react to MAID - Patients and families may feel disconnected from their religious community if it does not support MAID, resulting in social suffer   ***Practice Suggestions***   - Exploring the need for spiritual/religious counselling is important regardless of stated religious followings - It may be helpful to identify faith leaders from various religions who are supportive of MAID and can “step in” to the role of the patient/family’s usual faith community |
| **Offer organ donation, if potentially eligible** | *“*I think it’s a really good opportunity because most people, or a lot of people may feel that in choosing MAiD it’s a very selfish thing to do. And this sort of helps balance that” (Patient #421)  *Investigator:* And were you asked about your preferences for organ donation and if so, what did you think about that?  *Participant:* No I wasn’t asked that, but when I… When I renewed my license, I put on there that I would donate anything they needed. “(Patient #411) | - MAID patients were inconsistently approached for organ and tissue donation - Patients that were approached appreciated the opportunity to “give back”   ***Practice suggestions***   - Have a standardized screening process for donation so no more patients are approached than necessary, but also so that all potentially eligible patients receive the request - MAID assessor/providers may not be knowledgeable about donation; education may be required - Organ donation organizations should develop toolkits and standard practices to assist MAID assessors, providers, and patients with these discussions |
| **Process themes 4: Death and aftercare** | | |
| **Support patient choices for location, route, and timing of death** | “And so whatever their environment is and if somebody wanted to die at home then, you know…  But the family should agree that that’s what they want. Like the family should have some say because they have to live in that house” (Family member #212)  “Very peaceful and dignified way to pass..I think it’s enviable being the author of your own last chapter, as it were. Not everybody has that opportunity to make that decision. (Family member #912)  “Knowing that there is the possibility of me having a bit of control. I don’t have to have total control, but a little bit of control is still a little bit of control and, like I said, it allows me some form of comfort to say that, hey cancer, I have something to come back on. For want of a better word. It doesn’t have to be like that, but I just want to have the ability to have that small little amount of control.”(Patient #811)  “I talked to my daughters about it and they didn't think that it was a wise decision but when they came and I told them I had got permission to do this, they said… it's up to you mom, you're the one that's suffering. (Patient #821)  I don’t know. If we could mirror that team and across Canada. And even afterwards. Like they stayed afterwards and chatted with us and there was no…We were never rushed and [NAME] had said, you will never be rushed. So it was… It gave peace. That’s what it was. It gave peace. (Family member #812)  *Investigator:* How important is it to have control over your death? How important is that to you that that you've been able to… *Participant:* Oh gosh 100%. I mean I can't control the medical stuff and all that, but just knowing that this is what's in front of you. They’re not going to torture you; they’re not going to throw you under the bed somehow. This is how it all works. And I can't think of anything more ideal. (Patient #911)  *Do you think it gave your mom comfort to have that choice?* “My mother liked control, so I think for a control person, certainly. She controlled her life right up until the end. She had made her decision, and this is the way it’s going to be and I think that’s her… I think it was good for her. Not everybody’s going to be like that” (Family member #212)  *Investigator:* What’s the most important thing that the health care team can do to help a patient who's requesting MAID? *Participant:* Gently advising and letting know all of the ramifications, all of the possibilities. Is this a choice that you personally have made with your family and with the people around you? I think that's an important step. You can't fly alone, you know, you've got to have a support system. And I was luckiest woman in the world, because they all jumped in and said, Mom do what you want. We'll do it, we’ll back you up” (Patient #911)  “So we had a very tough talk one night and I said… let’s move forward because this is going to happen.... have you given any thought to when you want to do this and how you want to do it. So that was very difficult, but that’s a talk that had to take place. And I found once we had that conversation, she kind of went into a peace and I think somebody needed to say it to her because you get caught up in the visits...… Everybody’s human, you kind of go into denial. Nobody wants to leave this earth and leave behind people” (Family member #612)  *Do you have a preferred location?* “Too much logistics of getting home right now and we only live twenty minutes up the street … but I could have an accident on the way there, fall, you know, we don’t want any of that to happen” (Patient #811) | - Patients value choice over many aspects of their death: when, where, and who is present - Having a “set" date is often a source of relief and puts some patients at ease - Patients often feel that by choosing MAID they are sparing their family suffering - Families experience complex emotions; relief at the end of their loved one's suffering; grief at their death - Though patients might prefer a home death, many recognize and accept that the logistics may make hospital/institutional death an easier option   ***Practice suggestions***   - Provide clear information about feasible options regarding where, when, and how the MAID provision will occur - Provide the option of oral MAID provision to have further control only if available and feasible and provider is comfortable providing it - Explore with patients who they wish to have present during the provision; it may different from family preferences |
| **Choreograph the assisted death** | “He is the man for the job.....He knew exactly what he was doing and he knew exactly what to say and he knew exactly what to expect from people. We were totally happy and impressed with him. He just was totally on top of his game. He knew exactly, you know, what to say and do.  (Family member #712)  “It was almost like a wake that she was able to attend, an upbeat sort of a process.... That's how she really wanted to go” (Family member #912)  “It will happen here at the hospital and I already mentioned who I want there who I hope is going to be there and who I don’t want to see there, so she knows already.”   (Patient #231)  “It was his living wake and it was one thing that he always wanted to have. You know, so the hospital was… I know they bent rules for us which was also very appreciative, and they opened up their little room ... and gave us a place where people could go and sit and talk or if they weren’t in with my dad and… And so basically the day of is from 2:00 to 4:00 we had family and friends in. Which was surreal. … That was… That was tough. Because you’re putting a smile on in some respects, but you know that… You know, you’re kind of watching the clock. Because I knew basically at 4:00 I was going to have to kick everybody out and it would just be my mom, my dad and I” (Family member #512)  “I’ve been looking for Monday morning. They could only give me Monday afternoon, so that wasn’t absolutely perfect but I’m not complaining.... I wasn’t being shoved into a cue.” (Patient #411)  He put the needle in, and then he says, you just tell me when, it doesn't matter how long you take or whatever. Just tell me when you feel like you want to go. So she just laid there for a few minutes and we said a couple words and that. And then she said okay now and just went to sleep as if you were sitting there. And she went to sleep in front of you. Yeah. It was nice. It was nice. I can't believe how nice it was.  (Family member #622)  “So, being at XXX like I said, one room, one physician, and one team of nurses. It worked a lot better” (Family member #712)  “I think one of the reasons being that control over his life had been completely taken away from him. The situation he was in, he couldn’t leave there in the hospital, he was basically tied to machines. He was completely dependent on other people and he’s always lived by himself. He’s always been independent. So this was very, very hard for him. This gave him back the control he had lost over his life. (Family member #122)  *How important was it for your brother to have control over his death?* “Very important. That’s all he talked about in the end. Having control, it seemed that nothing else… He didn’t have control over anything else and he wanted it to happen on his own terms. Which was fine with us. We all accepted it and we didn’t question.”  (Family member #232)  “Well I think it was very important in the fact that he wanted this to happen and he didn’t want anybody trying to stop it... I mean the very fact that he asked for it in the first place, surely that in itself is a control really.” (Family member #432)  “I think it will be very peaceful for me too, because I want it so bad. I've wanted it for, I would say three or four months. I've been hoarding the pills” (Patient #821) | - Patients don’t want to feel that they are in a cue for provision - Patients and families appreciate feeling like the care they are receiving is “exceptional” - Despite this, many appreciate that MAID is logistically challenging within the constraints of provider availability, pharmacy, space, organ donation timeliness - Time can distort for patients and families; many watch the clock   ***Practice suggestions***   - Accommodate patient and family requests when feasible, but be honest when some options cannot be done - Take exceptional care to be on time or early as delays in provision are very distressing to some patients and families - Care coordination between locations can be logistically challenging and needs to be planned thoroughly and in advanced to ensure a smooth, confident provision process - Patients having control over their death is important to them - Dignity and independence through control brought by on MAID - Provide option for when where and how within reason for the provider to accommodate - Providers need to think about sustainability- not Friday at 7 if that is the only time available for family |
| **Prepare the family for the patient’s death and offer support afterwards** | Like do you just poke her and she dies? (Family member #822)  He came in and explained, you know, this is how it works. You do this and then we do this and then we do this, and then you just go to sleep. Sounds good to me. That's all I need. (Patient #911)  It was very beautiful to experience because it was very peaceful. They were very kind in how they explained what was going to happen. (Family member #612)  They sat beside her. He actually sat on the floor, you know, knelt down beside her on the floor and said, okay the procedure that is going to happen… And he explained the four different vials that he had and what they each did. And then he said to my mother, okay you tell me when you’re ready. And that’s when she said, yes I’m ready… The last vial he said um something that I didn’t think was necessary to say.... And then the last one he said, when I give you this one sometimes people, their bodies shake a bit ...I didn’t think he needed to say that to my mom” (Family member #322)  He made a noise after the first one that sort of all surprised us....Just a snoring noise sort of....That was a surprise to all of us. We sort of ah… like wasn’t sure what was happening. Maybe mentioning something like that, you know, could happen. Then you don’t feel so thrown off at that point. (Family member #232)  The only think I did talk to the doctor about is after it’s over and I’ve been declared dead is if they happen to walk by half an hour later, could they just check to make sure. (laughter) You know everybody’s eternal fear is you’ll end up in a box or something and be scratching on the inside. Nobody wants that! (Patient #411) | - Patients and families rarely know the specifics of the medical process and what an assisted death looks like - The transition of the patient from living to dead (pallor etc.) can be surprising to some family members - Some families wanted to know about the possibility of movement and sounds while their loved one dies and others were upset to learn   ***Practice suggestions***   - Tailor education to the needs and understanding of the patient and family - Ask how much detail families want to know; some value specific information on syringes, colour change, time until heart stops while others may not - Support and brief the family before and after provision - Having a space for patients to gather after and without being rushed |
| **5. Transcendent Theme 1: Coordination** | | |
| **Facilitate access** | I’m just so very glad that I was able to access it, I mean so very, very glad. In my case. (Patient #711)  *Is there anything that you’re most worried about?*  Just that others that don’t agree with the direction could interfere with it (Patient #421)  I’m just really again, very grateful that MAiD is now available in Canada. And congratulations to those who have made it possible. Because I think it is ah… a symbol of a compassionate society. Die with dignity. (Patient #141)  Let’s educate society and then it becomes a comfort instead of a resistance. (Family member #612)  I don’t see why anyone should be in charge of my life— also goes for my death. So I’m very independent in that respect. (Patient #631) | - Patients who are able to access MAID are very grateful the option is available to them - Patients and families perceive widespread stigma and resistance to MAID - Patients and families accessing MAID feels like a frustrating struggle despite it now being legal; this frustration is particularly acute as they view MAID as an expression of personal autonomy   ***Practice suggestions***   - Patients and families may require advocacy from clinicians to overcome barriers to access - Check-in on tasks and next steps to ensure the process continues to move along |
| **Provide continuity of care** | *If you could change anything about the process of requesting MAiD, what would it be?* Actually I found that easy. You know, it was simple. The wheels seemed well oiled. It was just went through… You know, the process just was very easy. (Family member #632)  They don’t come to you, which they should be part of that first meeting. You’ve got your radiation oncologist, you’ve got your oncologist, you should have a social worker there. Maybe that person could be the point person. Nurse. There needs to be someone from the MAiD team there....and that normalizes it then. (Family member #812)  It’s just that in the hospital they weren’t quite clear. What are the steps… exact steps...I think, really, even it was a well-documented printout that could be followed clearly …. a document that clearly indicates what are the steps that you need to take, these are the people that would need to be met. (Family member #132)  Once I was given the whole information leading up to the incurable cancer… I made up my mind right then and there. I said to the surgeon, I’m done. So then he proceeded to inform me that the MAiD program is here and available. So I said that’s what I want to do. (Patient #611)  *How important was it for your sister to have control over her death?* Very important....I mean if you could say it, that was sort of like a lifeline. That was like a… was a huge relief to her. She like to have control over things her whole life and her health has often been a thing that’s been her undoing. That’s prevented her from having control, so…she I mean obviously chose the day of her death, the time of her death, um and that was good. That was important to her. (Family member #412) | - Lack of care coordination is one of the most common concerns described by families - Care coordination services and organized MAID teams are helpful but the experience of getting access to those teams was often challenging - Many patients and families want to know that MAID is an option - Information needs to be given over time and by a trusted HCP   ***Practice suggestions***   - Ask the patient which members of their care team they would like involved in MAID - Engage the primary clinical team in the MAID process, including family physicians (if patient is in hospital) and sub specialists, irrespective of the patient’s location - Identify a “most responsible MAID clinician” or MAID coordinator to follow the patient |
| **Provide education and manage expectations** | I have to appreciate. I have to say, I never expected that level of kindness and cooperation, and information. It's like an information highway. You want to know something, we’ll tell you, just ask. It's a marvellous way because anything that involves the government, that doesn't usually happen. (Patient #911)  But I’d like to see more brochures or someone available. You know, there would be a number where you can call this program and they can explain a little bit more, or reach out to a counselor. Because, you know, you’re dealing with a very sudden death. (Family member #612)  It’s not a hurry, hurry, rush, rush process...It gives you time to think and, as I said, I don’t see anything wrong with the process. There might be some little tweaks here or there, but the basic… It’s just been a long damn time coming. (Patient #511)  I think the second thing is how they communicate with family. And again, my own experiences were, I never had to ask a question more than once. I was never searching for an answer. I knew that if I went to either one of them they were giving me the straight goods and that was all I needed. It didn’t always have to be good news but I was never left guessing either. (Family member #512)  My nurse practitioner.... made it very simple. She was able to give me a little bit more information at that point, I was still making decisions about whether it was going to be palliative or, you know, assisted dying. Once I heard all the details, I was pretty much sure this was the way I wanted to go. I asked about it, I was given an information package about it, and there was… I got a little confused. I thought there had to be ten days between the interviews. (Patient #411) | - Information needs vary within and between families, and over time - Families value the opportunity to have follow-up questions answered - Information overload is common, multiple check-ins and education sessions are helpful to ensure clarity around process - Patients and families value frank and transparent information and guidance around the process at every stage - Having a pamphlet with the steps outlined for reference was seen as helpful   ***Practice suggestions***   - Provide multiple opportunities and methods for education, written and verbal - Check for understanding - Ensure that patient and family expectations are clear with respect to eligibility criteria, what assisted dying can and cannot provide, and how flexible clinicians can be in providing assessments, assisting with preparation for death, provision, and aftercare |
| **5. Transcendent Theme 1: Patient centred care** | | |
| **Explore previous experiences with death and dying** | “I thought it was great. My feeling is someone’s suffering like that they shouldn't have to. I had my husband here. He was dying of pancreas cancer. I kept him home until he died. It's sad. It's sad. Just let them go. Let them go in peace... I would give it to anybody. I don’t care what anybody says” (Family member #622)  “I lost my husband about 10 years ago… He had an aneurysm and then during the surgery a stroke ... he lived eight more months of kind of a living hell ..... Just another aspect of healthcare that’s available now that wasn’t before. (Family member #412)  “I hope for a similar end someday, if I'm given opportunity. I think that was a real eye opening experience for all of us that were involved and everybody sort of felt like, you know, this isn’t a bad way to go, right” (Family member #912) | - Past experiences with death and dying influences how patients and families approach MAID - Having experience with death by withdrawal of life support in ICU was a common contrast to MAID   ***Practice suggestions***   - Probe for previous experienced with death and dying during assessments to help predict a patient and/or family’s needs throughout the process |
| **Preserve dignity and privacy** | And not only people that you love, but people you respect. Every woman and man who walks in here, I have the greatest respect for their integrity, their kindness. I can see it in their eyes. It's not… It's not pity. It’s ‘how can we help you?’ What do we need to do to make it better. (Patient #911)  *Investigator:* So what would quality care in assisted dying look like to you? *Participant:* It’s hard to say because the decisions that we made for [NAME] to be an organ donor. He didn’t have that comfort of being at home. It was in a clinical setting. But, in saying that, it was a very quiet… there was no one else on the floor but us and the team. So we could laugh and joke and play music and, you know, have a little chat with all the team members and they were all absolutely amazing....You did not feel like you were in a hospital because the team was so compassionate and warm and friendly, if you like. Like an old friend on the… even though we’d never met them before. You know, it was weird. It was really strange, but they were all so amazing. And I sent them  a letter to thank them all. No it was. It was amazing. The whole experience was. I mean as sad as you are, you don’t want them to go, but got to love them enough to let them go when that their time to go and I couldn’t have asked for anything better. I was really impressed with the whole process....we had quality care from the minute… So, in my experience, everything was fine. You know, we couldn’t fault any of the steps from the day he was diagnosed. You know, the ALS team at the hospital were amazing, very helpful. And the MAiD team, I don’t know how they do that on a regular basis. You know, I mean, I now the doctor said, you know, if he couldn’t have donated organs he would have come to the home and done it here which was wonderful. (Family member #632).  “And as I was saying, I believe mother nature has probably given me maybe two to three weeks at best anyway. And about the only thing I can come back with this is one small semblance of control that I have. Even if I can cheat death by a day, you know. And it might not be just cheating death, it might just be the fact that I have selected a day and this is where we’re at and I think everybody is in a good place and I just don’t want to have any more bodily accidents” (Patient #811)  Exactly. And they respected that privacy. They respected that nobody else was to know. You know, finally my dad did say to them okay, I’ve told my wife, you know, these family members know, so it’s no secret anymore. But I know they were very, very careful with that request that nobody knew for a period of time that it was just him and I. They were just…I mean they were phenomenal. They were just fantastic through the whole thing. (Family member #512) | - Patients and families described MAID as an intrinsically patient-centered act - The desire for the process to be centered around patient needs shapes the behaviour of many family members, and perceptions of the process - Many patients and families are sensitive to the possibility or stigma and value privacy - Patients and families often struggle to disclose the MAID request to friends and families   ***Practice suggestions***   - Provide compassionate care aimed at maintaining an individual’s dignity— what this means will vary between patients and families - Exercise even more than usual caution in keeping MAID information confidential than other personal health information |
| **Be sensitive to stigma** | And I said, well she called in a suicide and this is something that really needs to be dealt with in the public because they were talking on the news about that lady in Halifax and they referred to is as assisted suicide so many times I was disgusted. (Family member #612)  *Investigator:* And that’s I guess that last piece of, you know, not alone but also you’ve referenced it just a couple of times around stigma. Your mom didn’t tell everyone because she, you know… *Participant*: She didn’t know how people would react…. I don’t think my mother really cared if she got a negative reaction… But she didn’t want to hear it.  (Family member #212)  “Because it almost felt at times she had to defend her decision. And that’s going to happen no matter who it is, but it just… it would have alleviated a little bit of the stress for her. I tried my best to be present every day to sort of redirect the questions or be a support for her just so, you know, when those questions come at her… But I know there was times when even she called me at home and she’d be stressed and she’d say like, you know, can you come  up and tell people to stop calling me today because it’s the same questions over and over” (Family member #612)  So, for me, this part of the journey. I believe I’ve already reached that part through talking with family. I’ve had a conversation with one of my physicians here and, for him, ethically, religiously was a hard thing for him to do and he couldn’t condone it. But after talking with me, it has turned his head around to a better point of thinking that he would not want to endure this if it was this way. And all I can say is, I know it’s not the right thing. Try to put the shoe on the other foot. I’m a productive person, I like to meet people, I like to talk to people, I like to try and educate people and, at the same time I thrive on information and at the end of the day I will be receiving none of any of that.  (Patient #811) | - Stigma is experienced within the family; health care system and wider community at all stages of the MAID process - Patients and family have to continuously negotiate the stigmatization of others, defending or explain their choice to access MAID   ***Practice suggestions***   - Be aware that patients and families may have experienced stigma and may take time to trust even well-meaning clinicians - Anticipate that patients and families will struggle with whom to share information about MAID request and how/when to disclose; support patients to tell their families and friends - HCPs should explore with patients and their family members how they are going to tell and how they are going and offer support for those conversations |
| **Care for the whole family** | *Investigator:* What was the most important thing that the healthcare team could do to help patient requests when they…?  *Participant:* Look after my family. (Patient #131)  Is it the right thing for my family and do they understand it’s what I want....but going out with a smile because it’s what we ....I know it’s a hard way to go and all emotional suffering and that but just that they understand and they’re onboard with it. (Patient #221)  I'm fully behind this whole program, I hope that it has continued success and that a lot more people are able to take advantage of it...Yeah, it would have been nice to have another couple of weeks, maybe a couple of months even, who knows, right but it wouldn't have been any fun for her and she'd already made up her mind that she did not want any part of that I respected that decision and I was at peace with the whole process (Family member #912)  I think that perhaps counselling could be a bigger part. I was lucky I had it, but I don’t know what you provide for MAiD normally. I would think that counselling either for… also for family members. If my husband had say still been alive…I think he would have required counselling to… to face it. (Patient #141)  But she just did not think there was any quality of life worth living. She called us four siblings together and explained to us that she wanted to do this. That there was no point in going on and then she said, I want to hear all four of you, your opinions on it and if you’re all in agreement then I’ll talk to your dad. And that’s what happened. (Family member #322)  My journey. It’s the family’s journey, like I counted. (Family member #812) | - Thought patients are at the centre of MAID process, it has a dramatic impact upon families, who navigate complex emotional landscapes after their loved one's death - Comfort and caring to the family after their death is the biggest concern for many patients   ***Practice suggestions***   - Engage families early and if not involved, explore the reasons why with the patient - Anticipate the complexity of emotions that family members will experience supporting their loved one’s decision for a hastened death - Bereavement and follow-up services should be provided to families, where available |
